# Clinical Characteristics of Developmentally Delayed Children based on Interdisciplinary Evaluation

**DOI:** 10.1038/s41598-020-64875-8

**Published:** 2020-05-18

**Authors:** S. W. Kim, H. R. Jeon, H. J. Jung, J. A. Kim, J.-E. Song, J. Kim

**Affiliations:** 1 Department of Physical Medicine and Rehabilitation, National Health Insurance Service Ilsan Hospital, Goyang, Korea; 2 Department of Pediatrics, National Health Insurance Service Ilsan Hospital, Goyang, Korea; 3 Department of Psychiatry, National Health Insurance Service Ilsan Hospital, Goyang, Korea; 4 Department of Rehabilitation Medicine, Inje University Ilsan Paik Hospital, Goyang, Korea

**Keywords:** Autism spectrum disorders, Risk factors

## Abstract

The aim of this study is to examine the clinical characteristics of children suspected to have neurodevelopmental disorders and to present features that could be helpful diagnostic clues at the clinical assessment stage. All children who visited the interdisciplinary clinic for developmental problems from May 2001 to December 2014 were eligible for this study. Medical records of the children were reviewed. A total of 1,877 children were enrolled in this study. Most children were classified into four major diagnostic groups: global developmental delay (GDD), autism spectrum disorder (ASD), developmental language disorder (DLD) and motor delay (MD). GDD was the most common (43.9%), and boys were significantly more predominant than girls in all groups. When evaluating the predictive power of numerous risk factors, the probability of GDD was lower than the probability of ASD among boys, while the probability of GDD increased as independent walking age increased. Compared with GDD and DLD, the probability of GDD was increased when there was neonatal history or when the independent walking age was late. Comparison of ASD and DLD showed that the probability of ASD decreased when a maternal history was present, whereas the probability of ASD increased with male gender. To conclude, the present study revealed the clinical features of children with various neurodevelopmental disorders. These results are expected to be helpful for more effectively flagging children with potential neurodevelopmental disorders in the clinical setting.

## Introduction

Developmental disabilities caused by dysfunction of the central nervous system, including the brain, are called neurodevelopmental disorders, and children with neurodevelopmental disorders have difficulties in various fields including physical, linguistic, behavior and learning^1^. According to a previous study conducted in the United States, 5–17% of children suffer from developmental disabilities, and recent trends have shown a gradual increase^2^. Limitations due to neurodevelopmental disorders might continue throughout life, and individuals with these disorders may require special services, health care and support^3^. These factors cause enormous social costs to a country as well as economic and psychological burdens for the families of children with developmental disabilities^4^.

The cause of neurodevelopmental disorders varies, and it is difficult to distinguish between children with neurodevelopmental disorders and typically developing children in early infancy. Even if the neurodevelopmental disorder is caused by nonprogressive factors, the clinical phenotype may change over time as the central nervous system matures^5^. Therefore, children’s symptoms are different according to their age and severity, and the necessary interventions will vary accordingly. As a result, the diagnosis of a neurodevelopmental disorder can vary greatly depending on the clinician’s perspective, and the treatment or intervention or social support offered may differ according to diagnosis. The time at which an expert is consulted varies widely from newborn to school-aged^6^. As shown in previous studies^7,8^, intervention during the period when the brain is developing rapidly can minimize disabilities and reduce the gap in developmental delay; as such, it is important to start precise intervention early. Neurodevelopmental disorders express various features, and the degree of influence by developmental domain varies from case to case. Because of the multi-morbidity feature, attempting to intervene by focusing on only one problem can lead to not only overlooking other accompanying problems but also a problem of inefficient use of limited intervention resources.

To compensate for difficulties in dealing with the complexity of neurodevelopmental disorders, an interdisciplinary clinic named the Developmental Delay Clinic (DDC) has been operating in our hospital. In this clinic, three specialists (a pediatric neurologist, pediatric physiatrist and pediatric psychologist) work together to provide comprehensive diagnoses and intervention plans. The three specialists, depending on area of expertise, each examine children, prescribe necessary tests, share and discuss the results of physical and neurological examinations and various tests and produce a precise diagnosis with a balanced intervention plan for each child. In this study, the authors aimed to identify meaningful factors for diagnosis and to determine if it is possible to distinguish major neurodevelopmental disorders at the clinical assessment stage.

## Methods

Children who visited the DDC in our hospital with complaints of any developmental problems from May 2001 to December 2014 were included in this study. The total number of subjects was 1,877. Approval to perform this retrospective study was obtained from our Institutional Review Board (IRB) and research ethics committee (National Health Insurance Medical Center, NHIMC 2015-09-016). The need for informed consent was formally waived by the IRB and research ethics committee. All methods were performed in accordance with relevant guidelines and regulations.

All patients who visited the DDC for the first time had a history taken, and data were gathered according to the prescribed protocol. Data such as birth history, prenatal history, family history and other medical history were collected from a paper questionnaire. Birth history included intrauterine period and birth weight. Prenatal history included fetal distress, problems related to amniotic fluid or placenta, intrauterine growth retardation (IUGR), and fetal movement abnormality. Events such as fetal apnea, meconium aspiration and neonatal seizures were considered in the neonatal history. Postnatal history included infections such as sepsis, infantile spasm, and febrile convulsion. The presence of family history, such as language delay, autism spectrum disorder, and intellectual disability, and maternal history during the pregnancy period, such as anxiety or insomnia, depression, smoking and drinking, were also assessed in the survey.

**Figure 1:**
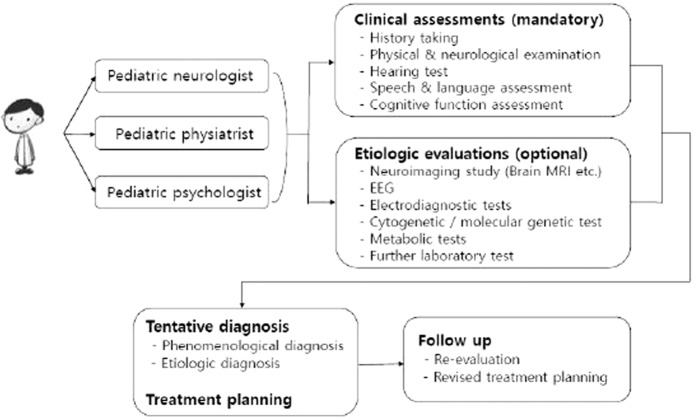
Diagnostic protocol for children visited developmental delay clinic.

After assessing histories through the questionnaire, the three specialists examined the child and prescribed necessary tests according to protocol. The diagnostic protocol was composed of two categories: required tests applied to all children and selective tests applied to some patients who needed those tests, based on each specialist’s judgment^9^ (Fig. 1, Supplementary 1).

The diagnosis was determined by discussion among the three specialists in reference to each child’s clinical findings and standardized developmental assessment results. The diagnoses were divided into two categories: either a phenomenological diagnosis based on the child’s current condition or an etiological diagnosis based on the pathophysiology of the condition. All these phenomenological diagnoses were classified into four major groups according to the child’s main features: global developmental delay (GDD), autism spectrum disorder (ASD), developmental language disorder (DLD) and motor delay (MD). The GDD group included diagnoses such as GDD and intellectual disability. GDD refers to children with significant delays in more than two of the following developmental domains: gross motor/fine motor, speech/language, intelligence, social interaction and self-care. In general, children under five years of age who met the requirements were diagnosed with GDD, while older children who could be examined using a reliable and formal intelligence test were diagnosed with intellectual disability^10^. Diagnoses such as reactive attachment disorder and social communication disorder were included in the ASD group. Those in the ASD group were diagnosed based on diagnostic criteria from the Diagnostic and Statistical Manual of Mental Disorders, 4^th^ edition (DSM-IV)^11^. However, since it has been updated from DSM-IV to DSM-V, the term ASD is used in this paper to prevent confusion. MD was defined as significant impairment of gross and/or fine-motor function compared with other developmental domains. Cerebral palsy and developmental coordination disorder were included in this group. DLD was defined as significant impairment of speech and language ability compared with other developmental domains. In this context, “significant” meant more than two standard deviations below the average value for the same age^10^. Etiological diagnoses included chromosomal and genetic anomalies, myopathy, and metabolic disease, among others.

### Statistical analysis

SAS ver. 9.2 (SAS Institute, Cary, NC, USA) was used for statistical analysis. The results of the survey were obtained using the Kruskal-Wallis test with Bonferroni correction and logistic regression analysis. The level of significance was set at p < 0.05.

## Results

A total of 1,877 children were enrolled in this study. When divided into classes according to major phenomenological diagnosis, GDD accounted for the largest number, with 824 children (43.9%), followed by ASD with 430 (22.9%), DLD with 389 (20.7%) and MD with 72 (3.8%). Only 16 children (0.9%) were finally diagnosed as developing normally after all tests and examinations were given. Boys were more predominant than girls, with 1,316 (70.1%) and 561 (29.9%), respectively (p < 0.05). The age at which children visited the DDC ranged from 2 months to 192 months, and the average age was 50.9 ± 30.0 months. The corrected age was used for preterm children until they reached two years old. Two hundred thirty-four children (12.5%) out of the total could be diagnosed with an etiological diagnosis. Among these, hypoxic ischemic encephalopathy accounted for the largest number, with 58 children (24.8%), followed by chromosomal and/or genetic abnormalities with 53 children (22.6%) and congenital anomalies of the brain with 33 children (14.0%). Among the children who underwent a brain MRI, abnormal findings were mostly found in MD with 27.8%, which was significantly higher than ASD and DLD (p < 0.05) (Table 1).

**Table 1:** Demographic data.

Variables		Number of patients (%)
Diagnosis	Global Developmental Delay	824 (43.9)
	Autism Spectrum Disorder	430 (22.9)
	Developmental Language Disorder	389 (20.7)
	Motor Delay	72 (3.8)
	Others	146 (7.8)
	Developing normally	16 (0.9)
Male	Global Developmental Delay	488 (59.2)
	Autism Spectrum Disorder	376 (87.4)
	Developmental Language Disorder	302 (77.6)
	Motor Delay	45 (62.5)
Age (months)	Global Developmental Delay	50.7 ± 30.2 (20.5–80.9)
	Autism Spectrum Disorder	51.0 ± 23.4 (27.6–74.4)
	Developmental Language Disorder	38.4 ± 16.3(22.1–54.7)
	Motor Delay	40.6 ± 38.7 (1.9–79.3)
Etiological diagnosis	Hypoxic ischemic encephalopathy	58 (24.8)
	Chromosomal and/or Genetic abnormalities	53 (22.6)
	Congenital anomalies of the brain	33 (14.0)
	Genetic muscle disorder	10 (4.3)
	Mitochondrial disease	4 (1.7)
Abnormal Brain MRI findings	Global Developmental Delay	106 (12.8)
	Autism Spectrum Disorder	11 (2.6)
	Developmental Language Disorder	5 (1.3)
	Motor Delay	20 (27.8)

With respect to preterm birth (gestational age less than 37 weeks), the history of preterm birth was the most prevalent in MD (29.2%), which was significantly higher than that in GDD (12.5%), ASD (10.9%) and DLD (8.7%) (p < 0.05). A history of low birth weight (LBW, birth weight less than 2,500 grams) was most common in MD (44.4%), which was significantly higher than that in ASD (20.9%) and DLD (25.4%) (p < 0.05) but not GDD (32.5%) (p = 0.426). Prenatal histories were most prevalent in MD (5.6%), which was significantly higher than in ASD and DLD (p < 0.05). Neonatal histories were also most prevalent in MD (29.2%), which was significantly higher than in the other three groups (p < 0.05). GDD and MD had a significantly higher prevalence of postnatal history compared with ASD and DLD (p < 0.05), but the difference between GDD and MD was not significant. Among family histories, language delay was the most common across all diagnosis groups, but the prevalence of having a family history did not differ significantly among the groups (p = 0.445). With regard to maternal histories, a maternal history of having anxiety or insomnia was the most common type in GDD, ASD and DLD, but drugs or drinking alcohol were the most common in MD. The percentage of cases with a maternal history did not differ significantly across the groups (p = 0.294) (Table 2).

**Table 2:** Risk factors related to neurodevelopmental disorders.

Variables		Number of patients (%)
Preterm birth	Global Developmental Delay	103 (12.5)
	Autism Spectrum Disorder	47 (10.9)
	Developmental Language Disorder	34 (8.7)
	Motor Delay	21 (29.2)
Low birth weight	Global Developmental Delay	268 (32.5)
	Autism Spectrum Disorder	90 (20.9)
	Developmental Language Disorder	99 (25.4)
	Motor Delay	32 (44.4)
Prenatal history	Global Developmental Delay	31 (3.8)
	Autism Spectrum Disorder	16 (3.7)
	Developmental Language Disorder	14 (3.6)
	Motor Delay	4 (5.6)
Neonatal history	Global Developmental Delay	145 (17.6)
	Autism Spectrum Disorder	56 (13.0)
	Developmental Language Disorder	57 (14.7)
	Motor Delay	21 (29.2)
Postnatal history	Global Developmental Delay	146 (17.7)
	Autism Spectrum Disorder	55 (12.8)
	Developmental Language Disorder	49 (12.6)
	Motor Delay	17 (23.6)
Family history	Global Developmental Delay	55 (6.7)
	Autism Spectrum Disorder	56 (13.0)
	Developmental Language Disorder	47 (12.1)
	Motor Delay	4 (5.6)
Maternal history	Global Developmental Delay	178 (21.6)
	Autism Spectrum Disorder	114 (26.5)
	Developmental Language Disorder	78 (20.1)
	Motor Delay	21 (29.2)

**Figure 2:**
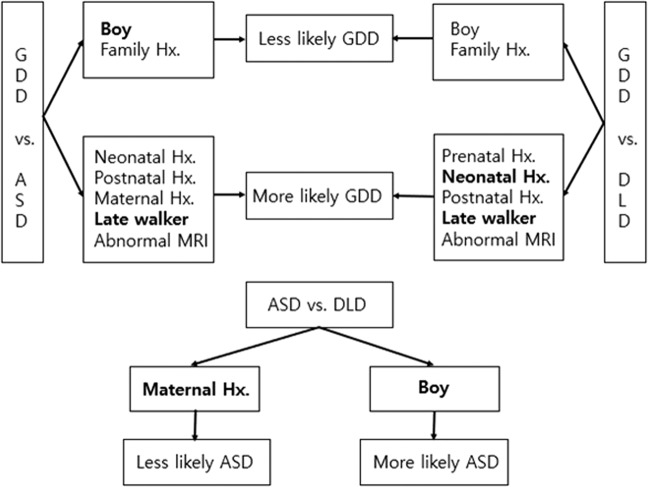
Distinctive clinical features among different diagnosis.

Among the various risk factors mentioned above, logistic regression analysis performed to compare the groups and to determine if certain risk factors contributed to being diagnosed with GDD, ASD and DLD. When comparing GDD with ASD, the risk of having GDD decreased with boys and the presence of family history, while the risk increased with the presence of neonatal, postnatal and maternal history, later independent walking age (a representation of delayed motor milestone) and abnormal findings in the brain MRI. After controlling for confounders, gender and independent walking age showed significant between-group differences. When comparing GDD with DLD, the risk of having GDD was lower in boys and with the presence of a family history, while the risk increased with presence of the prenatal, neonatal and postnatal history, later independent walking age and abnormal findings in the brain MRI. After controlling for confounders, neonatal history and independent walking age showed significant between-group differences. When comparing ASD with DLD, the risk of having ASD was higher in boys, while the risk decreased with the presence of maternal history. The results were the same after controlling for confounders (Table 3, Fig. 2).

**Table 3:** Multivariate Logistic regression model for prediction of diagnosis.

	*GDD vs. ASD*		*GDD vs. DLD*		*ASD vs. DLD*	
	*Odds ratio*	*95% CI*	*Odds ratio*	*95% CI*	*Odds ratio*	*95% CI*
Gender (male)	0.130^**^	0.060–0.282	0.581	0.289–1.166	1.906^**^	1.310–2.774
Prenatal history	—	—	1.431	0.437–4.678	-	—
Neonatal history	0.633	0.327–1.225	0.353^**^	0.170–0.732	—	—
Postnatal history	1.139	0.581–2.232	1.465	0.565–3.796	—	—
Family history	0.823	0.447–1.516	0.808	0.368–1.773	—	—
Maternal history	1.215	0.695–2.126	—	—	0.698^**^	0.499–0.976
Walking age	1.213^**^	1.127–1.306	1.184^**^	1.084–1.293	—	—
Abnormal MRI	1.825	0.788–4.230	3.455	0.999–11.950	—	—

^**^p < 0.05 on multivariate logistic regression, GDD; global developmental delay, ASD; autism spectrum disorder, DLD; developmental language disorder, MD; motor delay.

When receiver operating characteristic (ROC) curve analysis was performed to confirm the predictive power of these models, the model comparison of GDD vs. ASD and the model comparison of GDD vs. DLD showed good predictive power, while the model comparison of ASD vs. DLD had poor predictive power. Hosmer and Lemeshow’s Goodness-of-Fit Test revealed that all three logistic regression models were fit to predict the risk factors (Table 4).

**Table 4:** Predictive power of the logistic regression models.

	*AUC (95% C.I.)*	*p-value*
GDD vs. ASD	0.8045 (0.7607–0.8483)	0.4193^*^
GDD vs. DLD	0.7517 (0.6869–0.8166)	0.8971^*^
ASD vs. DLD	0.5664 (0.5327–0.6001)	0.7875^*^

*P > 0.05 on Hosmer and Lemeshow Goodness-of-Fit Test, AUC; area under the curve, GDD; global developmental delay, ASD; autism spectrum disorder, DLD; developmental language disorder.

## Discussion

The prevalence of developmental disabilities has risen in recent years with increases in high-risk pregnancies such as aged pregnancy, improved survival of high-risk infants due to medical technology advancement, and improved awareness and diagnosis of developmental disabilities^2^. The goal of early intervention for children with developmental disabilities is to prevent or minimize delays in all developmental domains, and early intervention allows children to achieve developmental milestones through the provision of enriched environments. Additionally, such interventions help caregivers cope efficiently with their children in daily life^12^. As seen in this study, the symptoms of children with neurodevelopmental disorders are very diverse, and the timing and symptoms of caregivers’ perception of something wrong in their children also vary. In addition, during the brain development period, one developmental domain affects the development of other domains, thus indicating multi-morbidity features. Proper intervention is important, but intervention is not always necessary. In some cases, it is more important to educate parents and modify the home environment than to use special resources. To effectively use limited resources, it is important to accurately diagnose neurodevelopmental disorders, which represent a multi-morbidity feature.

Among the patients who visited the DDC during the past 14 years, boys outnumbered girls in all diagnostic groups, which is consistent with previous studies^2,13^. Regarding etiological diagnosis, hypoxic ischemic encephalopathy was the most prevalent, followed by chromosomal and genetic abnormalities and congenital anomalies of the brain. These three factors accounted for 61.5% of the total etiologic causes. This outcome is similar to that of a study conducted by Shevell *et al*.^14^ indicating that four causes, i.e., the three causes mentioned above plus poisoning, accounted for 68.9% of total cases with a known etiological basis. There were no children with poisoning in the present study, which could be due to differences in socio-cultural backgrounds. However, more attention to antenatal poisoning might be needed, based on the recent increase in poisoning cases in Korea^15^.

In cases of preterm birth and LBW, which are known as the strongest risk factors for developmental disabilities^16^, a history of preterm birth was significantly more common in MD than in GDD, ASD and DLD. In contrast, a history of LBW was not significantly different between MD and GDD. It could be posited that the risk of GDD increased in cases of small for gestational age even in full-term births. Arcangeli *et al*.^17^ reported that compared with children of appropriate size for their gestational age, children who had a history of being small for their gestational age or who had fetal growth retardation, even in full-term births, showed lower neurodevelopmental scores. Takeuchi *et al*.^18^ reported that being small for gestational age is a risk factor for developmental disabilities, even in full-term babies. These results were consistent with the present study, and more attentive follow-up regarding developmental course is needed for children with a history of being small for gestational age.

Kumar *et al*.^19^ reported that the prevalence of neurodevelopmental disorders was higher in groups having family histories of neurodevelopmental disorders, such as epilepsy, GDD, MD, vision or hearing defects, compared with groups without such histories. Among the types of family histories, a history of language delay was seen the most in all diagnostic groups in this study. This finding could be explained by several factors: language delay is often present in various neurodevelopmental disorders, and the recognition and diagnosis of various neurodevelopmental disorders has improved in recent years, but this was not the case before. It may have been diagnosed as language delay^13^. In addition, it is possible that ASD has been diagnosed as other diseases, such as GDD or language delay, due to negative social perception of the diagnosis in Korea. Several studies have previously revealed that delay in one developmental domain often correlates with delay in other domains. Rechetnikov *et al*.^20^ stated that there was a correlation between motor impairment and speech and language disorder. Wang *et al*.^21^ reported that motor skill and communication skill were correlated with each other and that the motor skill of a one-and-a-half-year-old could predict the communication skill of a three-year-old. Language delay was predominant among the chief complaints of children who visited the DDC, but their final diagnosis was not limited to DLD. Shevell *et al*.^22^ reported that approximately three-quarters of children who were diagnosed with DLD before their fifth birthday showed some limitation of not only language but also communication, motor skill and social function at an early school age. Overall, the physicians would carefully assess all of the developmental domains, even if the chief complaints of parents were language delay, and would also give them a proper intervention plan focusing on the other domains.

This study has a few limitations. First, it is a single-center study, and most of the included children were from a metropolitan area in the Northern Gyeonggi territory. Second, children suspected to have cerebral palsy often visited the outpatient clinic of the rehabilitation department instead of the DDC for their initial evaluation, so the proportion of children with cerebral palsy was low in this study. Third, although the diagnosis may change over time, the study was conducted based on the initial diagnosis. Nevertheless, this study is meaningful in that it is the first study to present a probabilistic model in the clinical evaluation of children with suspected neurodevelopmental disorders. Several papers on the diagnosis of neurodevelopmental disorders that suggest diagnostic steps for GDD and ASD have been published thus far^23–27^. However, in contrast to the present study, there were no articles suggesting probabilistic models that included comprehensive history taking and clinical diagnosis. Additionally, most previous studies were confined to one diagnosis, such as cerebral palsy or intellectual disabilities, whereas this study represents the many children who visited interdisciplinary clinics for 14 years with various chief complaints about development.

In conclusion, the present study revealed the clinical characteristics of children who have developmental problems. In this study, we present a feature that can aid diagnosis in the stage of clinical evaluation for children with suspected neurodevelopmental disorders. These results are expected to be helpful for more effectively identifying children with potential neurodevelopmental disorders in the clinical setting.

## Supplementary information

supplementary 1.
