# Supplementary material for: Clinical Characteristics of Developmentally Delayed Children based on Interdisciplinary Evaluation
Source: Sci Rep. 2020 May 18;10:8148. doi: 10.1038/s41598-020-64875-8 (PMC7235222; doi:10.1038/s41598-020-64875-8)
Supplement: Supplementary file 1 — supplementary 1. [file 41598_2020_64875_MOESM1_ESM.docx]

**Title:** Clinical Characteristics of Developmentally Delayed Children based on Interdisciplinary Evaluation

**Running title:** Interdisciplinary Approach on Developmental Delay

**Authors:** S.W. Kim^1^, M.D., H.R. Jeon^1^, M.D., H.J. Jung^2^, M.D., J.A Kim^2^, M.D., J-E. Song^3^, M.D., J. Kim^4^, M.D., Ph.D.

**Affliations:**

^1^Department of Physical Medicine and Rehabilitation, National Health Insurance Service Ilsan Hospital, Goyang, Korea

^2^Department of Pediatric Neurology, National Health Insurance Service Ilsan Hospital, Goyang, Korea

^3^Department of Psychiatry, National Health Insurance Service Ilsan Hospital, Goyang, Korea

^4^Department of Rehabilitation Medicine, Inje University Ilsan Paik Hospital, Goyang, Korea

**Corresponding author:**

Jiyong Kim

Department of Rehabilitation Medicine, Inje University Ilsan Paik Hospital

Address: Juhwa-ro 170, Ilsanseo-gu, Goyang-si, Gyeonggi-do, Korea.

Phone number: +82-31-910-7885

Fax number: +82-31-910-7786

Email address: [halwayskim@gmail.com](mailto:halwayskim@gmail.com)

**Standardized test for clinical assessments**

Each test was chosen depending on the child's age and language abilities.

1. Speech and language assessment

- Sequenced Language Scale for Infants (SELSI)
- Preschool Receptive-Expressive Language Scale (PRES)

1. Cognitive function assessment

- Korean-Bayley Scales of Infant Development, 2nd edition (K-BSID-II)
- Korean-Wechsler Intelligence Scale for Children, 3rd edition (K-WPPSI-III)
- Korean-Wechsler Preschool and Primary Scale of Intelligence, 3^rd^/4^th^ edition (K-WISC-III/IV)
